# The Development of an Escape Room–Based Serious Game to Trigger Social Interaction and Communication Between High-Functioning Children With Autism and Their Peers: Iterative Design Approach

**DOI:** 10.2196/19765

**Published:** 2021-03-23

**Authors:** Gijs Terlouw, Derek Kuipers, Job van 't Veer, Jelle T Prins, Jean Pierre E N Pierie

**Affiliations:** 1 NHL Stenden University of Applied Sciences Leeuwarden Netherlands; 2 Medical Faculty Lifelong Learning, Education & Assessment Research Network University Medical Center Groningen University of Groningen Groningen Netherlands; 3 Research Group Serious Gaming NHL Stenden University of Applied Sciences Leeuwarden Netherlands; 4 Research Group Digital Innovation in Healthcare and Social Work NHL Stenden University of Applied Sciences Leeuwarden Netherlands; 5 University Medical Center Groningen University of Groningen Groningen Netherlands; 6 Post Graduate School of Medicine University Medical Center Groningen University of Groningen Groningen Netherlands; 7 Surgery Department Medical Center Leeuwarden Leeuwarden Netherlands

**Keywords:** serious game, autism, design research, boundary object

## Abstract

**Background:**

Children with autism spectrum disorder (ASD) have social deficits that affect social interactions, communication, and relationships with peers. Many existing interventions focus mainly on improving social skills in clinical settings. In addition to the direct instruction–based programs, activity-based programs could be of added value, especially to bridge the relational gap between children with ASD and their peers.

**Objective:**

The aim of this study is to describe an iterative design process for the development of an escape room–based serious game as a boundary object. The purpose of the serious game is to facilitate direct communication between high-functioning children with ASD and their peers, for the development of social skills on the one hand and strengthening relationships with peers through a fun and engaging activity on the other hand.

**Methods:**

This study is structured around the Design Research Framework to develop an escape room through an iterative-incremental process. With a pool of 37 children, including 23 children diagnosed with ASD (5 girls) and 14 children (7 girls) attending special primary education for other additional needs, 4 testing sessions around different prototypes were conducted. The beta prototype was subsequently reviewed by experts (n=12). During the design research process, we examined in small steps whether the developed prototypes are feasible and whether they have the potential to achieve the formulated goals of different stakeholders.

**Results:**

By testing various prototypes, several insights were found and used to improve the design. Insights were gained in finding a fitting and appealing theme for the children, composing the content, and addressing different constraints in applying the goals from the children’s and therapeutic perspectives. Eventually, a multiplayer virtual escape room, AScapeD, was developed. Three children can play the serious game in the same room on tablets. The first test shows that the game enacts equal cooperation and communication among the children.

**Conclusions:**

This paper presents an iterative design process for AScapeD. AScapeD enacts equal cooperation and communication in a playful way between children with ASD and their peers. The conceptual structure of an escape room contributes to the natural emergence of communication and cooperation. The iterative design process has been beneficial for finding a constructive game structure to address all formulated goals, and it contributed to the design of a serious game as a boundary object that mediates the various objectives of different stakeholders. We present 5 lessons learned from the design process. The developed prototype is feasible and has the potential to achieve the goals of the serious game.

## Introduction

### Background

Autism spectrum disorder (ASD) is a neurodevelopmental disorder. Children with ASD often face difficulties in initiating and maintaining conversations and find it challenging to interpret verbal and nonverbal behavior, which commonly leads to misunderstanding the intentions of others [1-4]. Children with ASD often do not spontaneously interact with peers and have difficulty making eye contact [5-7], often struggle to make friends [8], are more likely to be excluded by peers [8-15], and are more likely to be victims of peer harassment [16]. Many interventions developed for this target group focus on improving and training social skills [17]. One of the goals of social skill training is to enable these children to better cope with their peers. Although social skill training interventions demonstrate improved social skills in clinical settings, the developed social skills in training are not necessarily applied in children’s daily lives at school [18,19]. In addition, improving social skills does not necessarily mean breaking the negative bias of peers at school [20], which affects the quality and quantity of relationships.

The difficulties children with ASD face in forming and maintaining relationships with peers can lead to social fragmentation at school [21]. Research suggests a discrepancy in the quality and quantity of relationships reported by children with ASD compared with peers [22,23]. Some research even suggests that these children have the fewest friendships of all disabled groups [24]. In addition, according to the children themselves, they do not necessarily link how developing social skills contributes to forming more friendships and greater acceptance by peers at school [25]. Although one of the goals of social skill training is to better cope with peers, and social skills can improve in training, its effects do not always transfer to practice. Interventions that incorporate the peer-group context in natural settings can be beneficial for improving social skills [26] and improving relationships [27]. Nevertheless, there are very few interventions that focus specifically on this issue.

### Serious Games as Activity Context

Mutually enjoyed activities that focus on similar interests can be a fitting way for individuals with ASD to connect with others [28,29]. Therefore, activity-based interventions might be preferable for individuals with ASD than instruction-based interventions [28]. Activity-based interventions provide structure and a concrete focal point, allowing interactions to unfold more naturally [30]. Children with ASD seem to have an affinity toward digital technologies owing to their linearity and discreteness and often play video games themselves [25,31]. Several studies indicate that games can provide an enhanced experience compared with more common instruction-based interventions and teaching methods [32-34]. Games offer unique features to motivate, trigger, personalize, and facilitate learning [35,36] and provide a safe context for practicing more complex skills [37]. Furthermore, games can provide immediate and consistent feedback for children with ASD [38].

In autism research, there is great interest in the application of games to pursue therapeutic goals. Within the spectrum of games, a distinction can be made between interventions that gamify certain elements and interventions aimed at providing a good gaming experience. Interventions such as *LIFEisGAME* [39], *Secret Agent Society* [40], *Let’s Face It!* [41], *A Sunny Day* [42], and *TeachTown* [43] are based on a gamification approach, offering a reward system for finishing more traditional therapeutic tasks. This approach has 2 concerns: (1) although game elements and therapeutic tasks are combined, they do not provide a coherent experience [44], which is known as fidelity dissonance [45], an incongruence in in-game concepts that causes a disturbance in the game experience, and (2) compared with video games on the market, these interventions offer a poor game experience, which might undermine children’s interest in playing [44].

Interventions that facilitate collaboration and social interaction within a more integrated game design are *Invasion of the Wrong Planet* [46] and a technological touch-activated Collaborative Puzzle Game [47], offering an environment to collaborate through puzzling. *Pico’s Adventures* [44] is a game based on full-body interaction to promote social initiation skills. Another example of a serious gaming intervention is Lands of Fog [48,49]. Lands of Fog is a multiuser experience, designed with and for children with ASD to foster social initiation and collaborative behaviors. Within Lands of Fog, players can explore and discover unique characters, objects, and events in a magic world by catching fireflies. The game offers an immersive environment and provides specific mechanics to foster collaboration. This game appears to be a useful instrument to facilitate conversation and promising to promote engagement, socialization, and collaboration [48], which can positively affect children’s lives at school and their relationships. The game was able to unite children with and without ASD through an enjoyable and informal common activity–based experience. The practical disadvantage of Lands of Fog is that it is difficult to implement widely in terms of equipment and the required physical space. The system is structured in a 6-meter circular arena, where a virtual world is projected on a floor.

### Escape Room as Activity-Based Experience

Since its inception in Japan in 2007, escape rooms have been growing in popularity worldwide [50]. Entertainment-focused escape rooms are now available across most continents, including Europe, Asia, and America. In escape rooms, participants are given a scenario where they discover clues and solve puzzles to accomplish a specific goal in a limited time, usually escaping the room [51-53]. Participants often must work together and collaborate to fulfill different tasks successfully. Although research into the impact of escape rooms is still limited, escape rooms have already been used for various serious and learning-related purposes [54-57].

Due to the cooperative and game-like nature of escape rooms, escape rooms seem suitable for providing an informal activity–based experience for training social behavior and collaboration. Escape rooms are immersive and engaging [58], and the concept of puzzling and collaborating is fitting to integrate into a serious game. An escape game might offer a natural setting for players to communicate with their peers and can provide an emerging experience. This might allow interactions to unfold naturally. If properly designed, an escape game can offer a fun experience based on equivalent interactions and relationships, which is conditional on achieving friendships and acceptance among peers [27].

### A Serious Game as a Boundary Object

**Figure 1:**
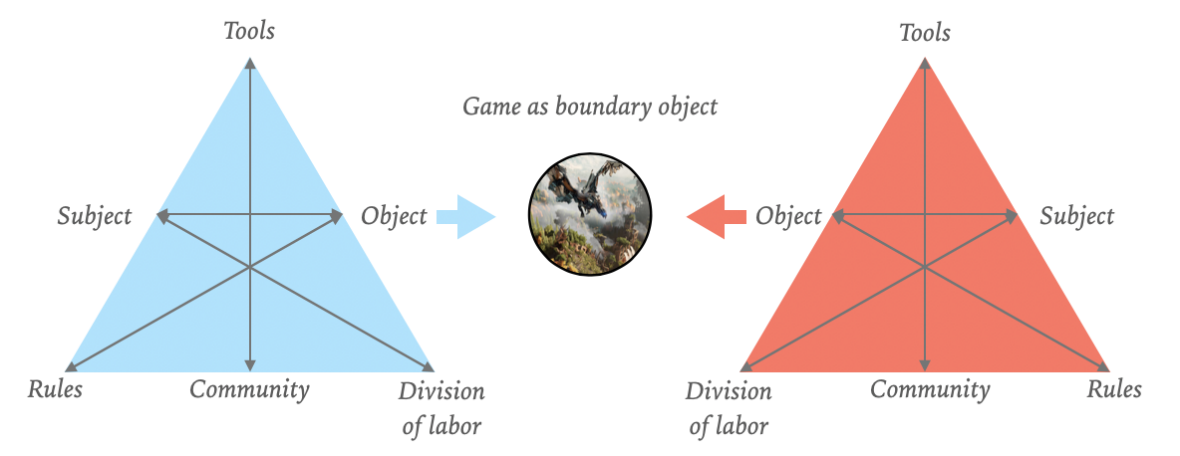
A game as a boundary object.

When designing a game with serious purposes, addressing the oxymoron and uniting the fun with the serious aspect is essential but challenging. Mapping out the various stakeholders’ perspectives and goals is often a necessary step because the interest of the fun element and the interest of the serious part is usually divided among stakeholder groups, especially when designing serious games for children. Experts who facilitate social skill training programs are interested in pursuing specific therapeutic goals. At the same time, children want to establish better relationships with their peers and, above all, seek a fun experience in a game. To overcome this lack of consensus, the concept of boundary objects can provide a perspective. The idea of boundary objects, which is growing in interest [59-63], was initially framed to facilitate constructive cooperation between sides or social systems in the absence of consensus [64]. Boundary objects [65] can fulfill an important function in overcoming boundaries by addressing and adapting the local needs and constraints of different stakeholders. As an in-between object, the boundary object belongs to the worlds of both stakeholder groups (Figure 1).

In the example of social skills training for children with ASD, children with ASD and experts who facilitate social skills training are connected and have a shared concern. Both parties are interested in making children function better in social situations; however, children have different goals than experts. Experts mainly focus on improving social skills, whereas children want to establish better relationships with their peers [25]. A well-designed serious game can be a novel platform to facilitate communication and a medium that strengthens relationships between sites [66] by creatively adapting and addressing user needs. Designing a serious game as a boundary object ensures that different stakeholders’ needs are addressed, which facilitates an inclusive design. The active inclusion of children’s goals and children with ASD is essential in designing a fitting serious game [44].

### Aim

This paper describes an iterative design process for the development of an escape room–based serious game. The purpose of the serious game is to facilitate direct communication between high-functioning children with ASD and their peers, for the development of social skills on the one hand and strengthening relationships with peers through a fun and engaging activity on the other hand. During the design research process, we examined in small steps whether the developed prototypes are feasible and whether they have the potential to achieve the objectives of the serious game. This paper aims to provide insight into the design process and describe the development of a serious game as a boundary object.

## Methods

### Study Design

**Figure 2:**
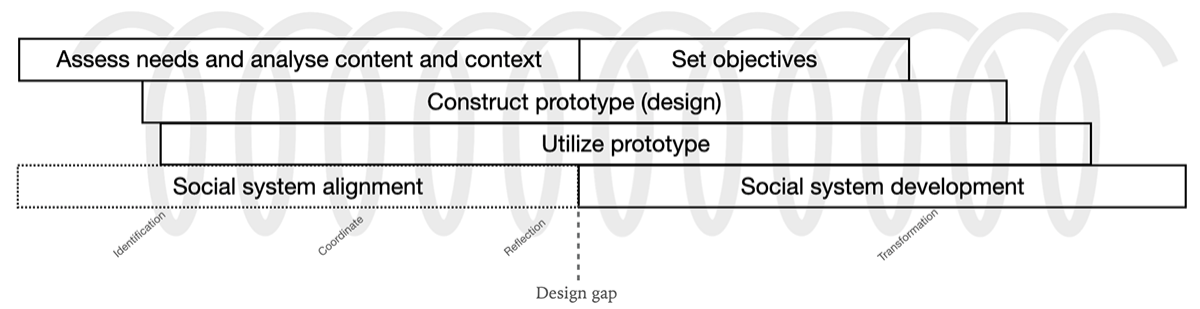
Design Research Framework.

This study is structured around the Design Research Framework (DRF; Figure 2) [67,68]. This framework facilitates the development of serious media interventions and serious games through an iterative-incremental process. The focus of these iterations shifts during the process, along with the nonlinear design steps [69].

The DRF gives direction within a design process on a more transversal level and can provide insight into where the focus in the development of an intervention lies. In our earlier research [25], we focused on assessing needs and analyzing content and context; this study focuses mainly on the construction and utilization of prototypes.

Various participatory design activities have been conducted to make the boundary object perspective applicable to the design process. As a boundary object, the serious game has to address both the therapeutic goals and needs as children’s needs while still being fun and engaging. Children with special needs can benefit from an inclusive design approach [70]. However, in practice, participation sometimes remains superficial and participatory design activities end up in sessions where children are mainly responding to, for example, visual aspects. Malinverni et al [44] proposed an inclusive design approach specifically for developing video games for children with ASD, where children and experts both provide input of specific elements that translate to game elements. Although the organization and structuring of sessions were slightly different in this study, this has been one of the central principles in the design process.

### Children’s Perspective

For children, forming more friendships, connecting with peers, and experiencing more acceptance by peers at school are important goals [25]. To achieve this, equality is considered an important principle. Equality is essential in forming relationships and friendships among children with ASD [27]. Further, mutually enjoyed activities that focus on similar interests can be a fitting way for individuals with ASD to connect with others [28,29]. A serious game that initiates social interaction in a structured way might be an appropriate way to address this issue. To arouse children’s and their peers’ interests, the game should also offer a fun and engaging experience. In addition, children provided input in finding an appealing theme and narrative for the game.

### Therapeutic Perspective

On the basis of an existing social skills training protocol, as described by Dekker et al [71], and literature on joint attention [72], 4 therapeutic goals have been taken as the guiding principles throughout the design process. These goals are turn-taking, cooperation, joint attention, and vocalization. The application and expression of these goals within the prototypes can be considered as the *early predictors of success* [73]. These principles guide the evaluation of the prototypes.

### Timeline

The sessions and activities described in this study took place within a 40-week school year, which runs from September to halfway through July. In this research, 4 iterations are described, consisting of developing and testing prototypes. The first 3 iterations were conducted within the first 20 weeks, always in a block of approximately 3 weeks. During the first 3 iterations, the authors developed the prototypes in 1-2 days and then tested the prototypes around schoolwork at various times within 3 weeks at schools. There were test periods and school breaks between the iterations, which made testing on location impossible. The final prototype was developed in 14 weeks by a professional development team, including the time required to form the team. This prototype was then tested with children and experts between June and July.

### Participants

#### Children

**Figure 3:**
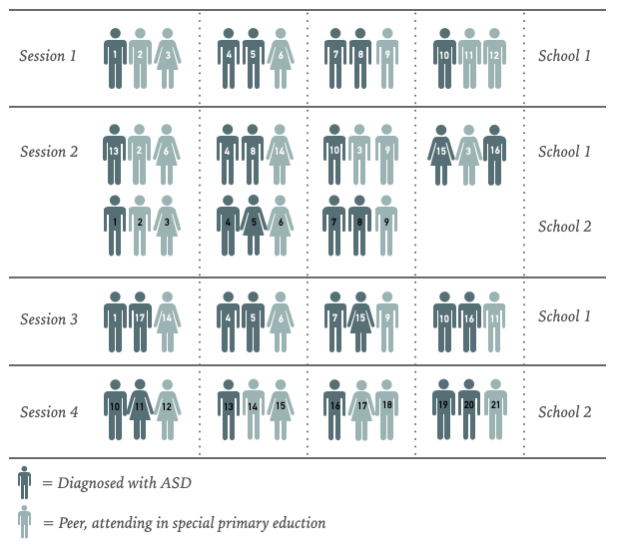
Participating children per session. ASD: autism spectrum disorder.

For the recruitment of the children, 2 special primary education schools participated. In the Netherlands, schools in special primary education have the same core objectives and curricula as that of regular primary schools. However, special primary education schools offer extra help to children with additional needs. The groups in special primary education are smaller, and there are more teachers and experts to assist the children. In the Netherlands, only children with additional needs attend a special primary education school. Those are children who are able to follow a regular primary school curriculum with some additional support, with the majority subsequently progressing to regular secondary education. In total, 3 classes functioned as pools for the sessions. Throughout the sessions, children diagnosed with ASD and their peers participated. The children with ASD have all been diagnosed with high-functioning autism. In total, 37 unique children participated in the study, 12 were girls and 25 were boys. A total of 5 girls and 18 boys were diagnosed with ASD. All children were aged between 10-12 years. Figure 3 shows an overview of how the pools’ participants had a place in specific sessions.

For considerations around privacy and ethics, more specific diagnostic characteristics of the children have not been documented, as have the other children’s medical and psychological backgrounds. For this research, which focuses on the feasibility of the prototypes to pursue specific goals, this personal information adds relatively little in this stage. The children and their parents provided informed consent. All retrieved data were processed anonymously.

#### Experts

A total of 12 experts were consulted to provide their input. The experts were connected to the project through participation requests within their organizations. The experts work in the field of child psychiatry (n=8) or at special primary education schools (n=4) and are familiar with the target group and regular social skill interventions for children with ASD. The experts provided informed consent. All retrieved data were processed anonymously.

### Evaluation 

In this study, 4 prototypes were tested in children. After the first test, a creative workshop was held to let children brainstorm about the theme of the escape room. We started with a paper prototype supported by materials from an existing escape room board game and ended with a multiplayer serious game playable on Samsung Galaxy Tab S2 tablets. On the basis of the different goals, the prototypes were evaluated after each test. Test sessions with children were also used to test the puzzles. The goal was to compose puzzles that were challenging and at an appropriate level for the specific age group of 10 to 12 years, by evaluating whether or not children can complete each puzzle given the information presented without any extra hints.

In the fourth test session, we also used the Playground Observation Checklist [74] to evaluate the play behavior. Although this checklist comes with some considerations and more background information on the children would be essential to make a reliable interpretation of the observation, the Playground Observation Test is a useful operationalization of social play behavior. In this phase of the study, the observation list was used to determine whether there were significant differences between playing behaviors among children playing the prototype.

## Results

### Overview

All tests have been carried out at the special primary schools. As there was always a larger pool of children present at the schools during the tests, there was no difference in the number of participants reported, as described in the *Methods* section.

### Iteration 1: Prototype 1 and Creative Workshop

#### Prototype

##### Prototype 1

**Figure 4:**
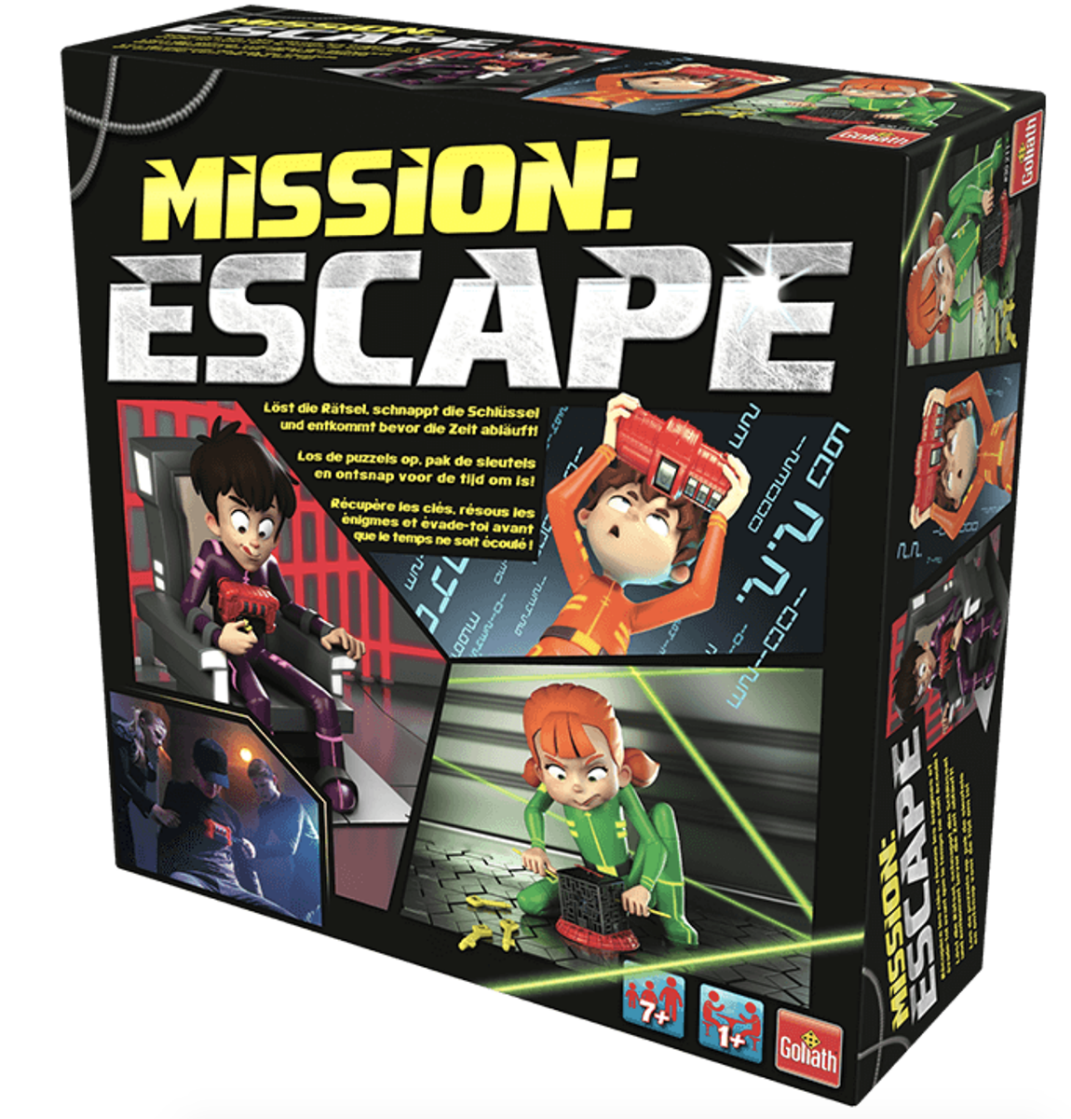
Board game—Mission: Escape.

**Figure 5:**
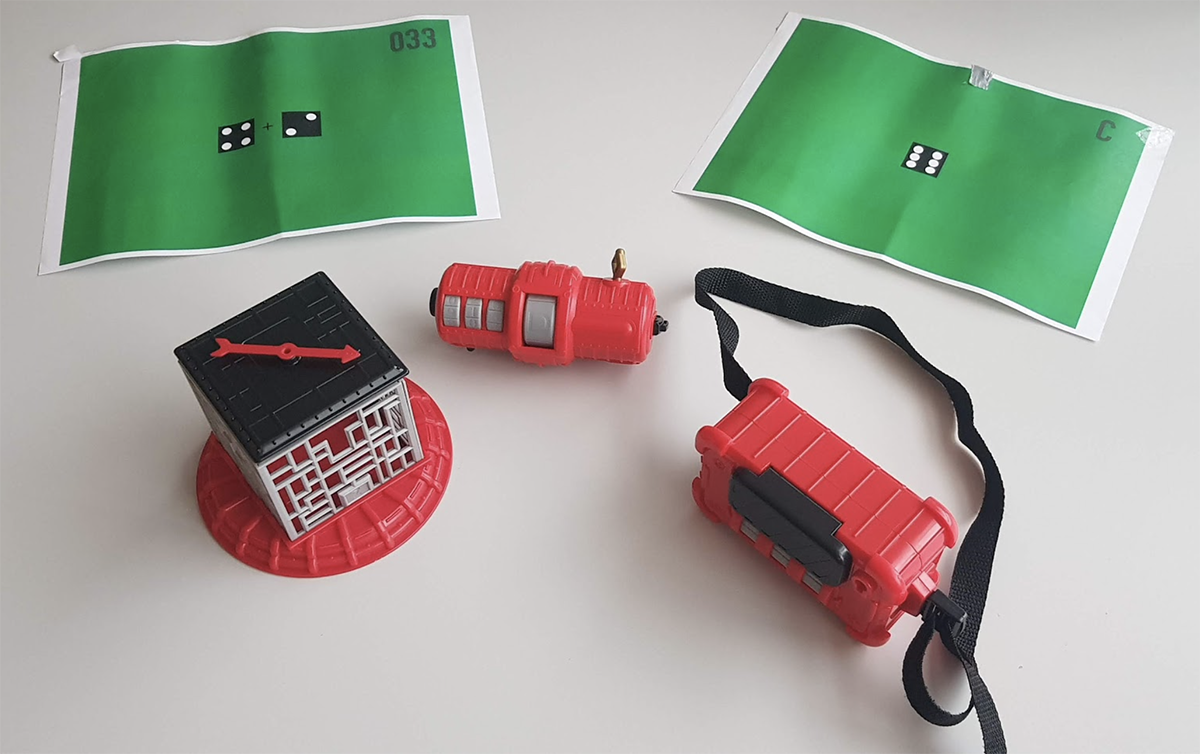
Materials of Mission: Escape.

For the first test session, an existing board game was used—*Mission: Escape* (Figure 4). This game is suitable for children aged 7 years or more and is based on the basic principles of an escape room concept. Within Mission: Escape, children must stop a timer with 2 keys before the predetermined elapsed time reaches 0. Children do this by retrieving 1 key out of a cage (Figure 5) and by retrieving a second key by solving multiple-choice riddles. The players can fill in the answers of the puzzles on a supplied artifact (Figure 5). After 3 correct answers in a row, the players obtain the second key.

**Figure 6:**
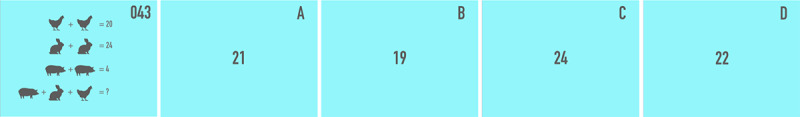
An example of a riddle.

For the test session, some adjustments were made to the game. Within the base game, the riddles of the game are presented on small cards. The base game can be played around a small table and is suitable for one or more players; however, the small cards do not encourage a collaborative game. Cooperation is an essential goal of the prototype developed in this study. To make collaboration more critical in gameplay, we separated the riddles and answers and printed them on small posters (Figures 5 and 6). By hanging the posters through the room in different spots, players must first connect the puzzle’s information to solve it.

##### Results: Test Session 1

During the first test, the mechanism with the ticking timer in all groups led to immersion and great motivation among the children during play. Children went straight to work and were motivated to solve the riddles as quickly as possible. The puzzles appeared to be solved relatively quickly for the children, and the children had only a little challenge in solving the puzzles. It took children, on average, 12 minutes to stop the timer. Although the puzzles were scattered in parts throughout the room, what should provoke cooperation, we noted that there was 1 child in all groups who claimed a very dominant role in the course of the game. This child took charge of the game and gameplay. The other children were involved in the game but more to locate specific answers—*Where is the red poster with a 4?*—and then from an equal level of influence to the game and gameplay. Although children seemed to have a fun experience and celebrated finishing the game together, one child’s dominance was not very constructive in pursuing different goals, especially turn-taking and equal cooperation.

#### Creative Workshop

After the play session, the children were asked to work out an idea for an escape room themselves, using drawing materials. Subsequently, each child briefly presented his or her concept. After the presentations, the drawings were put on the table, and the children could hand out stickers for the idea they liked the most. The children divided 6 stickers on maximum 3 designs. This workshop aimed to identify appealing themes for an escape room.

##### Results: Creative Workshop

**Figure 7:**
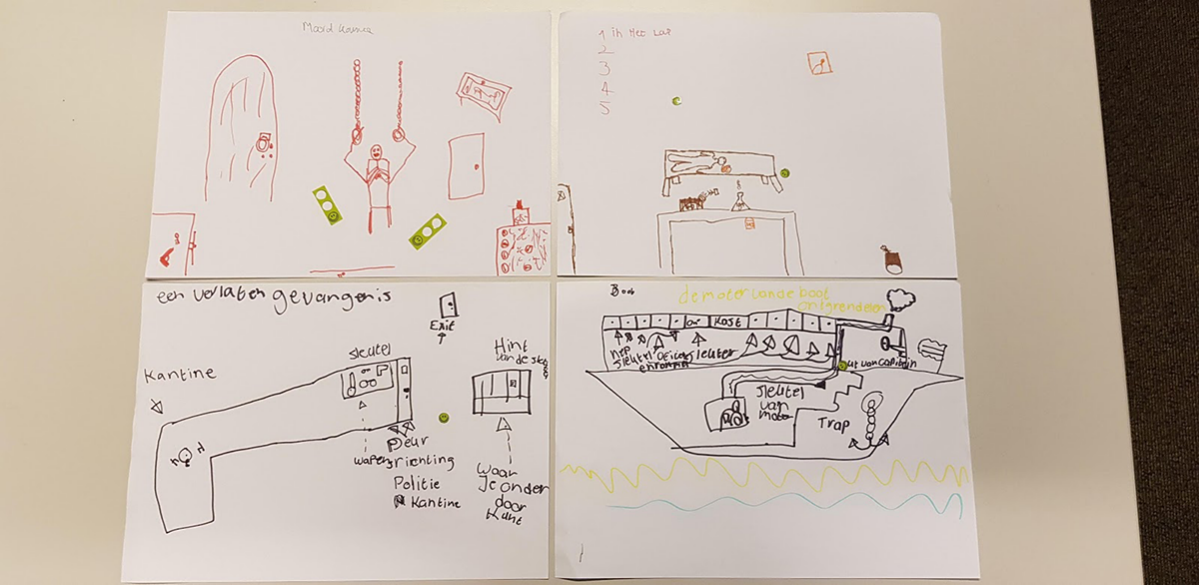
Impression of drawings in a creative workshop.

During the creative workshop, all children designed an escape room with drawing materials (Figure 7). The emphasis was primarily on the theme of the escape room that they would like. The setting of the drawings varied significantly; however, besides a boat, most children chose a darker theme, such as a dungeon, murder room, basement, chemistry laboratory, and prison. In the sessions with the different groups, these were also the drawings that received the most votes from peers, with the murder room and dungeon being the most popular. As an explanation, children explicitly indicated these themes that were the most exciting for an escape room. Or as one child put it, “from a room like this, you want to escape very quickly.”

#### Iteration 2: Augmented Reality Prototype

##### Prototype 2

**Figure 8:**
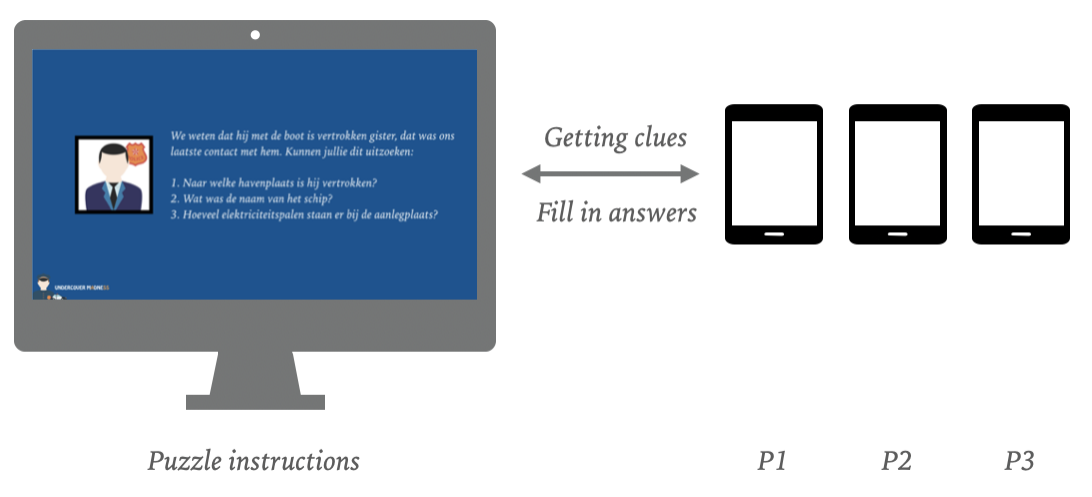
Setup for prototype 3.

For the second test, a simple prototype based on augmented reality (AR) technology was developed. The prototype is an app that deploys simple 3D models on real-world triggers. The app uses the camera’s input to present the real world and the layer with the 3D model simultaneously (Figure 8). The app was developed with Unity in combination with the Vuforia plug-in.

Next to the AR app, a simple web-based environment has been created, which presents the puzzles. During play, 1 player gets the main question of the puzzle; the other 2 players must search the correct 3D model to find the answer, for example, “How many floors do the two apartment complexes have combined?” The player with the web interface communicates the puzzle to the other players. The other 2 players search for an apartment complex, count the floors, and share their answers to the player with the web interface. The player with the web interface fills in the solution, and if the answer is correct, the next puzzle appears. During play, players swap their tablets twice. This makes sure that each of the 3 players is *the commander* in one stage of the game. The web interface communicates the moments for swapping the tablets. As each of the *scanning* players can only scan half of the posters, they are forced to work together.

The choice of the medium and structure of the game was the result of the experience from the first iteration. By distributing the information divided among the tablets, the players are more dependent on each other and have to collaborate and exchange information more, which appeals to their collaboration and turn-taking skills. The goal of this test was to evaluate whether the players would collaborate more if the essential information was divided among the tablets and whether the players had an equal share through changing roles. For this prototype, we composed new puzzles, and the test was used to assess whether the children understood the puzzles.

##### Results: Test Session 2

The 3D models that the children could see through the AR technology caused many positively surprised shouts in the first phase of the game, for example, “Wow, this looks cool!” In all play sessions, the children spent the first 3-7 minutes to scan as many triggers as possible to discover all the different 3D models. When there was nothing left to check, the focus of the children shifted to solving riddles.

The game sessions mostly started with a good balance in cooperation and communication, where all children had an equal role. However, after a while, when the game advanced, the applied mechanism again resulted in a situation where 1 player became increasingly dominant. In all sessions, this was the player with the riddles on his or her tablet, and it manifested itself in the later stage of the game. As the second and third children who received the tablet with the puzzles had previous knowledge of where specific models were located, those children happened to become more and more direct and commanding in their communication. It did not matter whether this was a child with or without ASD. The fact that the children could walk freely in the room reinforced this. It often happened that the player with the tablet with the riddles dragged other children along to different corners of the room to guide them to the 3D model belonging to the puzzle. Although the children were very motivated to solve all puzzles and were happy when they reached the end of the game, the deployment of mechanics to enhance turn-taking and equal cooperation during the whole game was still something to improve. However, it was better in the first phase of the game than in the playtests of the first prototype.

#### Iteration 3: AR Prototype With External Puzzle Instruction

##### Prototype 3

**Figure 9:**
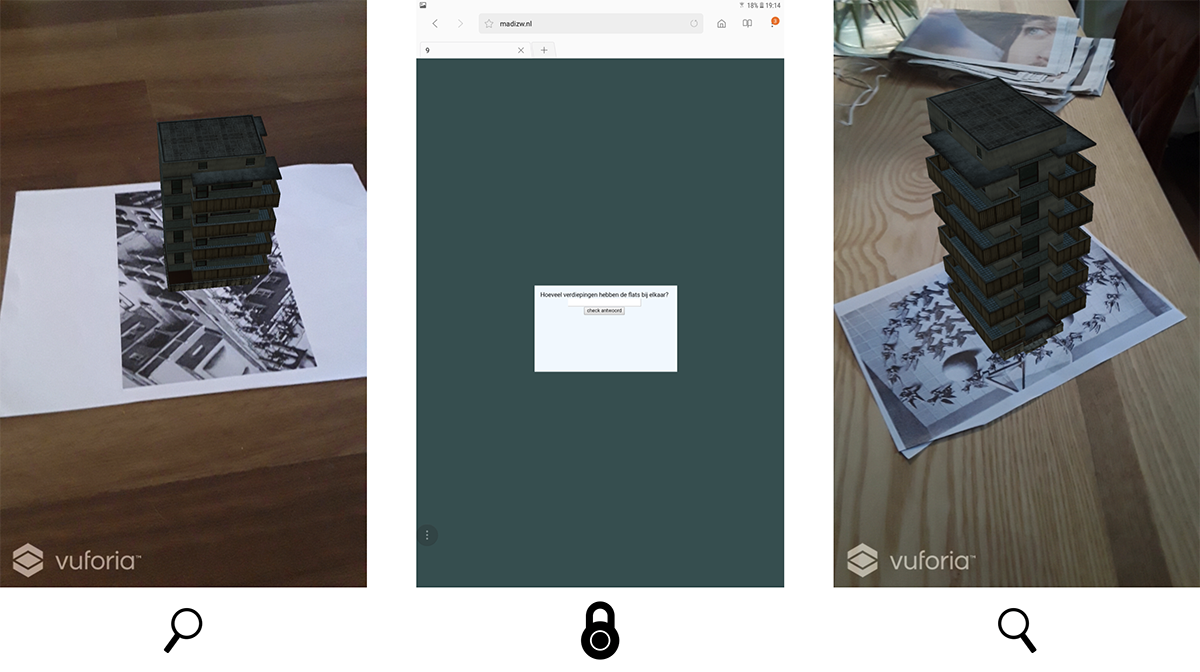
Augmented reality prototype.

The third prototype was based on the same principles and technology as that of the second prototype. The AR app operates in the same manner. The web interface was replaced by a television screen that presented the riddles using a laptop with a keynote presentation (Figure 9). As, during the previous iteration, the cooperation started in an equal manner but was later disturbed by the insider knowledge of players who later became commander, in this prototype, it was decided to have the puzzles presented by a neutral medium.

After a short story that introduces a scenario, the game starts. The story was introductory to a scenario in which children were asked to reconstruct a missing undercover agent’s timeline and eventually find the agent’s location. Within this prototype, all 3 players are searching for 3D models, and the players do not have to exchange tablets during the game. When the players have found the clues belonging to the puzzle and have the answer to it, one of them stands in front of the television screen and speaks out the answer. A moderator checks the answer, and if the answer is correct, he or she will ensure that the next riddle is presented. The triggers in the third prototype were distributed over the 3 tablets, which meant that each player had precisely one-third of the necessary information to solve the puzzles. The main goal was to check whether the new mechanics led to a more equal play among the players.

##### Results: Test Session 3

The narration turned out to provide extra focus and motivation for the children. It was found that the children were well immersed in the story. After one session, which could not be fully completed because of time constraints, 1 of the children came back to ask if it had all worked out well in the end for the missing policeman.

The new mechanism of offering riddles ensured more balanced cooperation. None of the children became very dominant during play in the different game sessions. However, the mechanism also resulted in sessions where children often worked side by side instead of with each other. As the riddles were presented to all players at the same time, less exchange and communication was needed. Each player read which part of the puzzle he or she could solve; searched for the 3D model belonging to the puzzle; and then, only, in the last step, while sharing his or her part of the answer, came to interact with the other children. This led to a very task-oriented collaboration. Turn-taking took place more naturally in terms of goals, and the players had a more equal role in the game. However, there was hardly any communication during cooperative play.

#### Iteration 4: Beta Prototype and Evaluation

##### Beta Prototype

**Figure 10:**
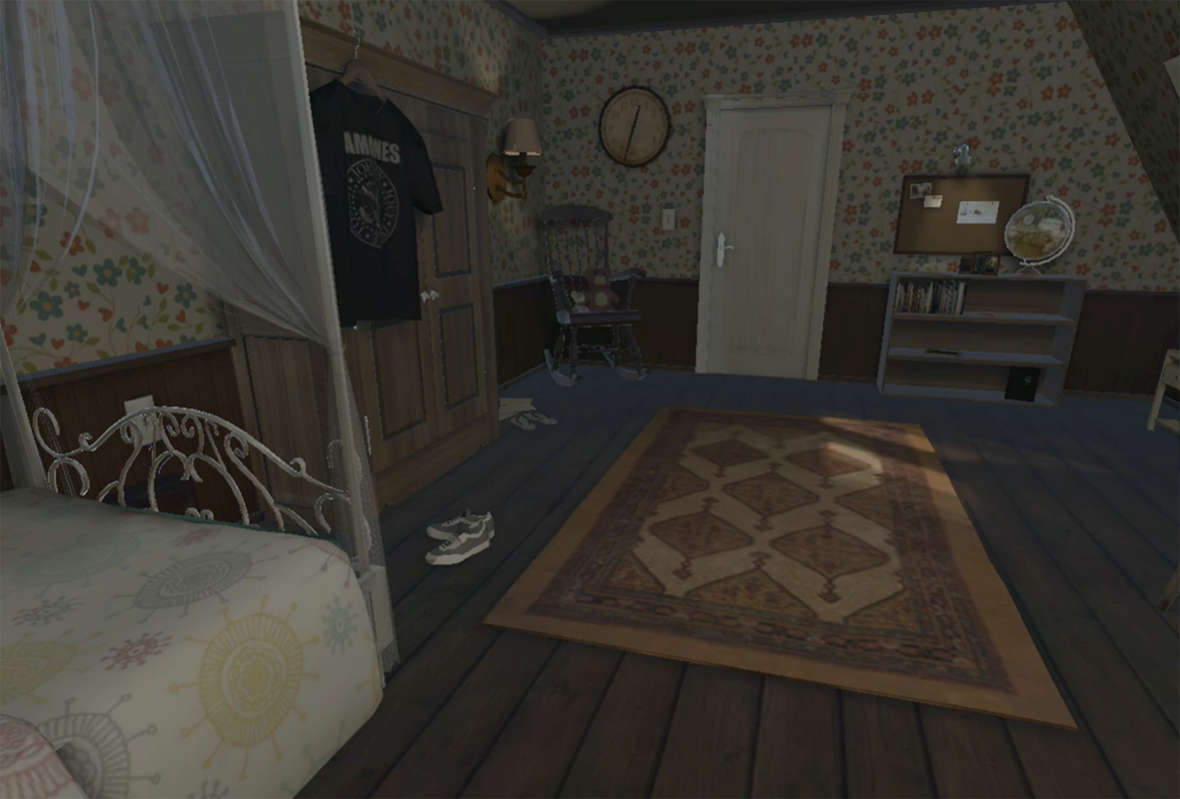
In-game screenshot of AScapeD.

**Figure 11:**
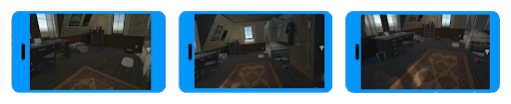
A 3-player setup of AScapeD.

For the last session, the beta prototype of a serious game named AScapeD was developed (Figure 10). Within the game, the players work as detectives on the case of a missing girl named Charlotta. The players are introduced into the case with a short story, and then they are placed in the girl’s room. All the rooms have the same appearance; however, each room’s time is different for each player; therefore, there are various objects placed in other places. By solving riddles and bringing together information from different rooms, players advance through the game. On the basis of previous iterations’ experiences, we decided to create a digital room because it makes it easier to create 3 unique rooms, each with their own pieces of the puzzle (Figure 11). This ensures that children have to collaborate and prevent a child from becoming dominant in the game based on previous knowledge. The design also ensures that the children have an equal role; each player is equally important in completing the game.

Most puzzles for this prototype are constructed so that each time another player gets the first clue, often by a blinking object in the room. This player needs to catch up with the other players by describing what they see because such specific information usually contains one or two clues for the other players to find the information belonging to the puzzle to solve it. This way of constructing the puzzles triggers collaboration skills, turn-taking, and vocalization. For the 3 puzzles, the object begins to blink simultaneously in all the rooms. These 3 puzzles are based on classic games. Within 2 of these games, players take turns within the game. These are a mastermind game and a classic car sliding puzzle. The third game, a classic sliding puzzle, is solved by each player individually.

During the game, players find 3 fragments from the girl’s diary: one from the morning, one from noon, and one from the early evening. Players learn that Charlotta feels a little bit lonely at school and does not connect very well with her peers. Eventually, she would run away to the cottage of her grandpa in the forest because her grandpa is always helpful and understanding.

The choice for a digital version of an escape room is to keep the context of use flexible. We found out with earlier prototypes that there is a practical constraint in the spaces we could use. Many special primary schools do not have the facilities to set up a larger room for an intervention for a more extended period. For extra support or interventions within these schools, you often have to use small offices. The 3 tablets make the context of use flexible: all you need to apply the serious game is the 3 tablets and 3 stools for the children, on which the children can physically rotate. A digital version also makes it easier to disseminate essential information among players.

The game is a result of various insights obtained from both theory and previous field tests, which translated into the final design. In addition to the design choices linked to the goals, as described in Table 1, more specific decisions were made to create the serious escape game. The creative workshops with children inspired the chosen theme and the story, where the children unanimously chose thrilling themes for the game. However, the choice was made to translate the degree of urgency to progress through the game into a narrative, something that has added value in escape room design [58], rather than a terrifying theme such as a murder room that potentially contains repulsive images. For the girl, an archetypical figure was chosen that faces similar challenges in daily life as children with ASD [25]. In a debriefing session, children can together reflect that children sometimes feel alone in the classroom and introduce peers to the challenges some children face at school. The puzzles and their levels are a result of the 3 previous test sessions and the process of testing and reconstructing them. An overview of the puzzles can be found in Multimedia Appendix 1.

**Table 1:** Goals and translations into design.

Perspective and goals		Translations into design	Legitimation
**Children**			
	Fun and engaging	Appearance Challenging puzzles Exploring as game mechanic Escape room structure for engagement	Creative workshop with kids (iteration 1) Test sessions prototypes Discovery as game esthetic [75] Conceptual escape room structure as an engaging mechanism [58]
	Connection with peers	Activity-based game Narrative transportation Narrative to give input for debrief Equal roles during play	Theory on activity based [30] Narrative transportation [76] An archetypical figure was chosen that faces similar challenges [25] Equality as a mechanism in forming relationships and friendships [27], thoroughly tested in previous test sessions
	Theme and narrative	Thrilling atmosphere Storyline for the escape room	Creative workshop with kids (iteration 1) Narrative as an essential ingredient for escape rooms [58]
**Therapeutic**			
	Turn-taking	Necessary information to fulfill game divided over tablets	Distribution divided among players, as a result of previous test sessions and theory on information distribution [44]
	Cooperation and equality	Integration cooperative mini games Roles different per puzzle	Cooperative nature of escape rooms [54-58] Results from previous test sessions
	Joint attention	Necessity to coordinate attention between game and puzzles and the other players	Choice for medium based on the results from previous test sessions and theory on information distribution [44]
	Vocalization	Distribution and presentation of information puzzles lead to the necessity to verbalize information until the communicative goal is met	Distribution divided among players and choice for medium, which facilitates this mechanism, as a result of previous test sessions and theory on information distribution [44]

##### Results: Game Test

Before the test session, the children received a short briefing explaining the game’s controls and stating that cooperation and communication would be essential for the successful completion of the game. At the beginning of the session, the children expressed their appreciation of the game’s graphics. The children’s experience with games ensured that the children quickly learned how to operate the game.

While playing, the players mostly spent the first few minutes exploring their rooms. Sometimes, a player had already started on a first puzzle but did not yet get in touch with his or her fellow players, because he or she was still busy looking around in the room. All groups entered the flow of the game after about 3 minutes, and from that moment on, they started communicating. The game requires the players to continually switch between looking at the tablet and communicating and looking at each other face-to-face. In practice, this mechanism did not hinder face-to-face contact very much. Children had a moment of dialog at each puzzle during play sessions, where they lowered the tablet to take a moment to discuss and exchange information. Generally speaking, the input on collaboration seemed equal throughout the sessions; each player was more in control at certain times in the game because of the composition of the puzzles and the distribution of information.

After about two-thirds of the game, the game contains a puzzle in which all players have to search for a part of a numerical code with an infrared flashlight. As this puzzle’s flow is deliberately different (the only puzzle where 3 players have to look for a clue and exchange the object), players often got stuck here for some time. One group could not get past this puzzle by itself; the other groups needed a small hint to get back on track. This puzzle put the players’ patience and skill of turn-taking to the test. At the end of the game, the players celebrated their success together. Play sessions lasted an average of 40 minutes.

On the basis of the test, the puzzles seemed neither too easy nor too difficult, given that the children only needed one small hint to get back on track during the game. The children were focused and immersed during the game and knew based on the feedback—every puzzle was a step closer to the end of the game—that there was progress in the game. The goals of the activity were clear given that the children started immediately and asked no questions during the game. After the game, when the facilitator told how long it had taken the children, children were often surprised that the time had gone so quickly, indicating that they had lost some of their sense of time during play. According to one of the children: “I didn’t realize we had been playing for so long.”

During the game, the children were observed using the Playground Observations checklist. During the game session, the researcher scored 10 items as either present or absent. The researcher did not know which children were diagnosed with ASD during the observation. Afterward, a teaching assistant who was always present during the test checked the observation scores. The results are presented in Tables 2 to 4. The results show no significant differences in play behavior between children diagnosed with ASD and their peers who attended special primary education for different needs. Most of these behaviors were observed. After the game, the children rated their cooperation level and gave a rating to the game. Only one child rated the level of cooperation relatively low, with a 7 out of 10. All children gave a positive rating for the game.

**Table 2:** Participants’ game test results.

Item	Participant											
	1	2	3	4	5	6	7	8	9	10	11	12
Gender	M^a^	F^b^	F	M	M	F	M	F	M	M	M	M
Diagnosed with ASD^c^	No	Yes	No	Yes	No	No	Yes	No	No	No	Yes	Yes

^a^M: male.

^b^F: female.

^c^ASD: autism spectrum disorder.

**Table 3:** Results: Playground Observations checklist.

Item	Participant											
	1	2	3	4	5	6	7	8	9	10	11	12
Engages in social play with peers	1^a^	1	1	0^b^	1	1	1	1	1	1	1	1
Is not socially isolated from peers	1	1	1	0	1	1	1	1	1	1	0	1
Respects boundaries and personal space	0	1	1	1	1	1	1	1	1	1	1	1
Does not exhibit socially inappropriate behavior	1	1	1	1	1	1	1	1	1	1	1	0
Follows rules of a game	1	1	1	1	1	1	1	1	1	1	1	1
Responds to winning or losing	1	1	1	1	1	1	1	1	1	1	1	1
Initiates communication with peers	1	1	1	1	1	1	1	1	1	1	1	1
Sustains a conversation with a peer	1	1	1	1	1	1	1	1	1	1	1	1
Does not exhibit gross motor incoordination	1	1	1	1	1	1	1	1	1	1	1	1
Uses playground equipment functionally	1	1	1	1	1	1	1	1	1	1	1	1

^a^1: present.

^b^0: absent.

**Table 4:** Participants’ ratings.

Item	Participant											
	1	2	3	4	5	6	7	8	9	10	11	12
Score (out of 10)	8	10	9	8	10	10	10	10	10	10	9	9
Rating level of cooperation (1-10)	9	8	8	8	7.5	8.5	10	9	9.5	8	7	9
Rating game	9.5	9	9	9	9	9	10	10	9.5	9	9.5	9

#### Expert Evaluation

For the expert evaluation, a session was organized at a central location for all participating experts who normally work from different locations. During the session with the experts, first, the game was played. The experts had more trouble with the controls; however, once they had managed to control them, they were at least as enthusiastic and immersed as the children. The experts did not go through the whole game because of limited time but could get a clear picture of the game.

Experts indicated that the game could have added value to their work because it gives them an immediate impression of communication between children. They also see many possibilities in manipulating the puzzles, for example, to test patience or build up frustration in a controlled way and to reflect on this with the children subsequently. All experts indicated that the game was particularly suitable for children who already knew each other to some degree, such as children from the same class.

The experts recognized the different social skills that are usually embedded in social skill training:

Actually, there are many skills and sub-skills in the game, which is normally dealt with in social skills training. But they are triggered more naturally, as we saw for ourselves when we were playing.

Upon further questioning, it appeared that the experts were referring to turn-taking, verbalizing specific images on the screen to the other players, and switching between playing and talking. Furthermore, the experts mentioned that each child plays an essential role in the game, as they take turns with a vital piece of information. However, some social skills trainers indicate that specific skills must already have been trained:

I doubt if you can use it directly, it does require a certain basis.

At the same time, they see merit in trying the tool at several locations:

...at home with a brother and a parent, for example. You may be able to observe other patterns that influence communication.

All experts indicated that they wanted to use the game in their practice. Experts from child psychiatric institutions would like to use the game in the final phase of group therapy so that children can put all the social skills they have learned into practice. Experts from special primary schools would like to use the game more freely and see potential in the game to develop group dynamics at the beginning of the school year. They indicate that developing the skills included in the game might add value to all children in the class. Effective cooperation, dividing attention well, and waiting for turns can increase the classroom’s synergy. They would also preferably use the serious game for a more extended period and with multiple levels to prolong the experience and formulate goals with the children in a debrief and follow-up about what they can do differently next time. Special primary school experts also see a lot of potential in a good and entertaining storyline. The story remains alive between the sessions: “Children of this age are sensitive to stories, especially when certain things in the story have yet to be unravelled.”

## Discussion

### Principal Findings

In this study, we designed AScapeD, a serious game to facilitate social interaction and communication between children with ASD and their peers. AScapeD contains various elements of existing knowledge and practical insights resulting from an iterative design research process. AScapeD was deliberately designed to provide a useful activity-based experience for players. This might be an appropriate way for the target group to directly execute social skills in a safe context. The serious game is designed to be played with children with and without ASD, as this can contribute to socialization [48], can trigger peer support [77], and might increase the likelihood of transfer [45] by allowing the children to play directly in the target context.

The game has been developed and tested in small steps to ensure that it offers an immersive and meaningful experience. There was a focus on achieving a fun and challenging experience during these steps by finding the puzzles’ appropriate level of challenge for children aged 10-12 years. There was also a focus on narrative transportation [76] to involve the players over a more extended period. An escape room’s conceptual structure contributes, even in digital form, to the natural emergence of communication and equal cooperation among children. The iterative process has contributed to translating the therapeutic goals into the game in a constructive way. It was a challenge to construct a game in which communication was necessary. Players had to draw on their ability to take turns, and everybody had to have an equal role throughout the game. Everyone was in the lead for approximately the same amount of time. By using 3 rapidly constructed prototypes, each of which took less than 2 days to complete, it was possible to quickly learn which translations from goals to game mechanics were effective.

On the basis of the first results, AScapeD appears to be a promising serious gaming tool to successfully trigger social interaction and connection in a playful way between children with ASD and their peers. While playing, children with ASD participate actively and equally. During play, the play behaviors of children with and without ASD did not differ significantly. Experts recognized the skills of turn-taking, vocalization, joint attention, and the necessity for cooperation in the game. The serious game appears to be feasible to pursue the formulated goals; however, according to the experts, the children already need to be at a certain base level to apply those skills. Further research should focus on what base level is required. For effectiveness, no conclusions can be drawn yet based on the observations and the applied scale.

### Research Process

AScapeD results from an iterative design process structured around the DRF [67,68]. The DRF gives direction within a design process on a more transversal level, where constructing and improving prototypes in an iterative way is a central guiding theme, which was also a leading principle in this study. The DRF provides focus and direction on the central design process; however, other resources are necessary to address specific audience characteristics and mechanisms to pursue specific goals. In this study, we could quickly learn from prototypes to find a fitting and constructive translation of predefined goals into the serious game. In addition, it has been of added value to actively involve the children in the entire process and incorporate their goals and wishes in the serious game. By approaching the serious game as a boundary object throughout the entire design process, trying to address the local needs of different stakeholders, there was a constant focus on creating an inclusive design.

Involving children with a particular vulnerability in the research process is always a challenge. Nevertheless, in human-computer interaction design, it is usual to actively involve end users in a design process. Inspired by the inclusive design approach of Malinverni et al [44], we have followed this in this study by enabling children to trigger their strengths and interests and letting them play the prototypes of the serious game and let them respond to it and let them give input on specific design choices. This made it easy for children to get quickly involved in the process and made the input and feedback questions on the different prototypes very tangible.

**Figure 12:**
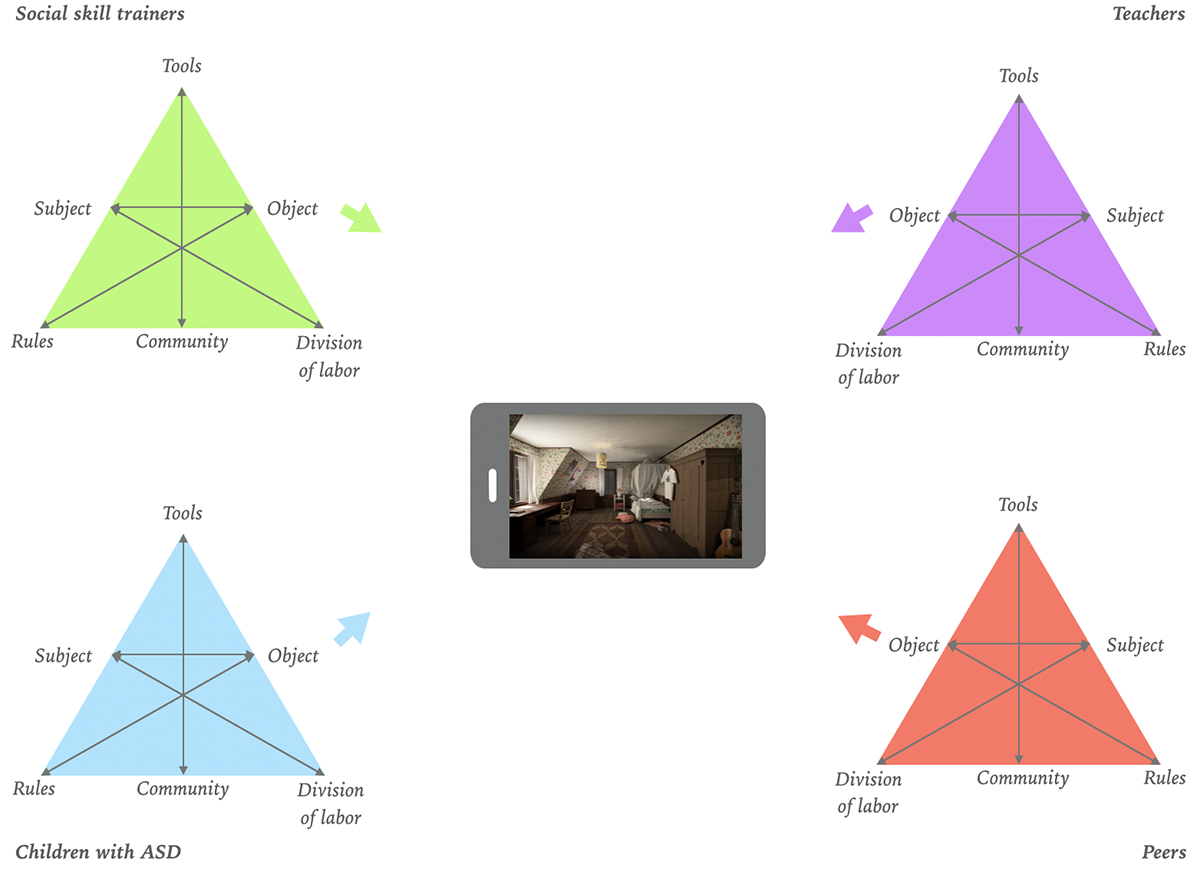
AScapeD as a boundary object. ASD: autism spectrum disorder.

As a result of approaching the serious game as a boundary object, the process has not led to a new tool for a specific activity system. The applied process has led to a mediating tool that contributes to different involved activity systems objects without attempting to achieve consensus between them (Figure 12). AScapeD adapts the various stakeholders’ local needs and constraints and obtains a different meaning from the various activity systems. For children with ASD, a serious game is a tool that contributes to their goal of better connecting with peers. For social skills trainers, it is a tool that allows them to see how children with ASD put social skills into practice. For special primary school teachers, it is a tool that can be of added value for the development of group dynamics. The skills included can be beneficial for each child. Turn-taking, joint attention, and cooperative skills can be beneficial for each child and the group dynamics in a classroom, even if there is no developmental issue such as with children with ASD. In conclusion, for peers of children with ASD, it is a tool to have a fun experience and establish relations with peers.

### Guidelines for Future Work

From this study, we identified 5 important insights that could be useful as guidelines for future work. The following suggestions emerged from this study:

1. An iterative design approach helps to constructively translate different goals into a game. Prototyping and an iterative approach are common in game design [78-80]. In this study, this approach helped us to find the right translations from goals to game mechanics. By constructing and testing rapidly built prototypes, we obtained feedback on how constructively the predefined objectives were translated into game mechanics and how and whether the children experienced the game as a fun experience.
2. Approaching the serious game from the beginning as a boundary object [65] is helpful in addressing and adapting the local needs and constraints of different stakeholders. This perspective eventually resulted in an inclusive design. Considering the game as a boundary object has led to a continuous focus on different user needs.
3. To promote collaborative behavior, it is advisable to distribute the necessary resources, as we found out in testing different prototypes. This insight is in line with advice from previous research that included cooperative mechanisms in a game for children with ASD [44].
4. An escape room’s conceptual structure is useful for enacting communication and collaboration in a digital environment. The learning potential of escape rooms has been increasingly acknowledged in the literature [54-58]. The findings of this study indicate that the conceptual structure is also applicable to a digital environment.
5. Involving children and experts is an added value when developing a game. Inspired by the inclusive design approach of Malinverni et al [44], we gave both groups a specific role in the process. Children have provided more input on specific playful experiences and experts on therapeutic goals. In this study, this approach ensured a structured and purposeful process.

This study’s inclusive approach offers a specific perspective for shaping innovation in health, mainly when innovation affects multiple activity systems. Many frameworks on implementation in health have been developed within disciplines [81]. These frameworks are, therefore, very suitable for innovation within an activity system to improve its tools. Innovation in more complex contexts involving multiple stakeholders often leads to high failure rates or a lack of impact [82]. The boundary object perspective focuses on bridging the gap between different goals among stakeholders.

### Limitations

For the development of the serious game, different groups of participants participated in the study. The serious game seems promising based on the first results. However, further research is necessary to investigate whether the game is applicable in practice and whether the serious game is of added value in the longer term. On the basis of this study, the game seems suitable for high-functioning children with ASD, given that they could actively participate in the game, had an active role in solving the puzzles, and could actively communicate and collaborate during the game. The puzzles’ level is tailored to children who have the IQ to follow a regular school curriculum. Scalability is, therefore, more likely to occur toward application in other contexts, where the experts call the application in a home setting and schoolteachers, for example, the application in different classes to strengthen group dynamics, rather than toward application to other target groups with, for example, children with a different diagnosis of autism or a lower IQ.

More long-term research with the serious game will have to show whether the serious game can fulfill its potential in the longer term. In a follow-up study focusing on effect rather than feasibility, the correct background variables need to be mapped out more closely. In addition, the application of a measuring instrument requires more consideration.

### Conclusions

This paper presented the iterative design process of AScapeD, a serious game based on the concepts of an escape room. AScapeD triggers social interaction and connection in a playful way between children with ASD and their peers. An escape room’s conceptual structure contributes to the natural emergence of communication and cooperation between children within a fun and engaging activity. AScapeD results from an iterative design process, where many insights were gained by learning from the application of rapidly constructed prototypes in practice. Applying prototypes contributed to finding a constructive translation of children’s goals and therapeutic goals into the serious game. Children were actively involved in the study by participating in playful test sessions that triggered their strengths and interests by letting them play the serious game prototypes and allow them to respond to it. By approaching the serious game as a boundary object throughout the entire design process, attempting to address the local needs of different stakeholders, there was a constant focus on creating an inclusive design.

## Appendix

Multimedia Appendix 1 Overview of puzzles.

## Abbreviations

AR: augmented reality
ASD: autism spectrum disorder
DRF: Design Research Framework
