# Supplementary material for: The Development of an Escape Room–Based Serious Game to Trigger Social Interaction and Communication Between High-Functioning Children With Autism and Their Peers: Iterative Design Approach
Source: JMIR Serious Games. 2021 Mar 23;9(1):e19765. doi: 10.2196/19765 (PMC8294642; doi:10.2196/19765)
Supplement: Multimedia Appendix 1 [file games_v9i1e19765_app1.pdf]

Lobby / Awaiting all players are ready

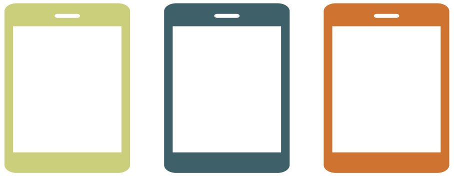

Tab

Narrative

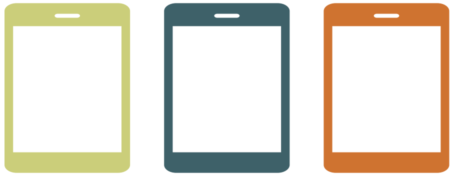

Tab

Players proceed to room

Setup:

Players enter the same room, but at different times. Within the puzzles, they bring together objects / things / information from different times, in order to progress and open locks. Ultimate goal: find out what she wrote in her diary of where she went.

Clock: 08:21

Travelbag: empty

Diary not in open space

Clock: 12:33

Travelbag: packed

Diary on chair

Clock: 16:47

Travelbag: gone

Diary on table

Window open

Pre-puzzle events:

Trigger, diary blinks

Hmmm, locked. Possibly this diary will tell you where she went, the key to this diary might be the key to the answer.

Puzzle 1:

Drawer desk blinks<br>*Tab*<br>Numeral lock + Two symbols<br>+		
	In the room, lable with 2 numbers	Number on bottom
*Unlock		
*Key in drawer		

Puzzle 1 events:		
		P3 can examine locker with lock.<br>(Travelbag blocks the locker for P1 / P2)<br><br>P3 can open locker.<br><br>Players have to find out that they can pass on items.
		Case with two keyholes.<br>*Tab*<br>To inventory

Puzzle 2:

	Trigger, drawing bulletin board blinks<br>↓ *Tab*<br>Drawing with rebus / sum of missing numbers	
P1 counts symbol x number of times		P3 counts symbol y number of times
	With counting, sum can be solved. Answer 9.	
	*object with 9 can be opened (hatch in calendar.)<br>1 key for case	

Puzzle 3:

Trigger, mastermind game blinks	Trigger, mastermind game blinks	Trigger, mastermind game blinks
Players solve mastermind, in turns	Players solve mastermind, in turns	Players solve mastermind, in turns

Puzzle 3 events:		
		Mastermind gives note with code
Drawer lights up > code opens drawer<br>Drawer contains diary morning version<br>Story		

Puzzle 4:

Switch at a hatch lights up.
Wires are loose, switch does not work.

Hmm, there seems to be a clue behind this, we will just have to fix the switch first. But how?

Somewhere in room, screwdriver

Somewhere in room, wire socket

Once socket + screwdriver are at player 2, player 2 can fix the switch.

Puzzle 4 events:

	Hatch open, infrared flashlight in inventory.	

Puzzle 5:		
Locker lights up		
Players take turns using infrared lamp to search for code in the room.<br>3	5	8
Safe can be opened with correct combination		

Puzzle 6:		
Safe contains drawing, pointing to a switch by the bed.		
		Player can find switch, with a number lock next to it.
		Turning switch causes a certain light bulb to pulsate for all players.
7 x	5 x	6 x
		Code 756 opens the lock

Puzzle 6 events:		
	Correct code leads to a light spot in a dark place where the diary lies.<br><br>Story	
Photo grandfather lights up.<br><br>Investigations lead to a bill with arrows and crosses, a kind of treasure map without a map.<br><br>> In inventory		

Puzzle 7:

Trigger, car slidings puzzle blinks	Trigger, car slidings puzzle blinks	Trigger, car slidings puzzle blinks
Players solve car slidings puzzle, in turns	Players solve car slidings puzzle, in turns	Players solve car slidings puzzle, in turns

Puzzle 7 events:

		Reward, key for diary<br><br>Story

Puzzle 8:

Trigger, sliding puzzle blinks	Trigger, sliding puzzle blinks	Trigger, sliding puzzle blinks
Players solve sliding puzzle individual	Players solve sliding puzzle individual	Players solve sliding puzzle individual

Puzzle 9:

Reward, part of the map	Reward, part of the map	Reward, part of the map
Players should combine maps		
		Player 3 can escape through window<br><br>Triggers end of the game, final story
